# Colon-Targeted Poly(ADP-ribose) Polymerase Inhibitors Synergize Therapeutic Effects of Mesalazine Against Rat Colitis Induced by 2,4-Dinitrobenzenesulfonic Acid

**DOI:** 10.3390/pharmaceutics16121546

**Published:** 2024-12-02

**Authors:** Changyu Kang, Jaejeong Kim, Yeonhee Jeong, Jin-Wook Yoo, Yunjin Jung

**Affiliations:** College of Pharmacy, Pusan National University, Busan 46241, Republic of Korea; whale10000@naver.com (C.K.); wowjd9669@naver.com (J.K.); jyhsyh0518@naver.com (Y.J.); jinwook@pusan.ac.kr (J.-W.Y.)

**Keywords:** poly(ADP-ribose) polymerase inhibitor, colon-targeted drug delivery, colitis, mesalazine, prodrug

## Abstract

**Background/Objectives:** In addition to oncological applications, poly(ADP-ribose) polymerase (PARP) inhibitors have potential as anti-inflammatory agents. Colon-targeted delivery of PARP inhibitors has been evaluated as a pharmaceutical strategy to enhance their safety and therapeutic efficacy against gut inflammation. **Methods:** Colon-targeted PARP inhibitors 5-aminoisoquinoline (5-AIQ) and 3-aminobenzamide (3-AB) were designed and synthesized by azo coupling with salicylic acid (SA), yielding 5-AIQ azo-linked with SA (AQSA) and 3-AB azo-linked with SA (ABSA). Additional conjugation of AQSA with acidic amino acids yielded glutamic acid-conjugated AQSA (AQSA-Glu) and aspartic acid-conjugated AQSA, which further increased the hydrophilicity of AQSA. **Results:** The distribution coefficients of PARP inhibitors were lowered by chemical modifications, which correlated well with drug permeability via the Caco-2 cell monolayer. All derivatives were effectively converted to their corresponding PARP inhibitors in the cecal contents. Compared with observations in the oral administration of PARP inhibitors, AQSA-Glu and ABSA resulted in the accumulation of much greater amounts of each PARP inhibitor in the cecum. ABSA accumulated mesalazine (5-ASA) in the cecum to a similar extent as sulfasalazine (SSZ), a colon-targeted 5-ASA prodrug. In the DNBS-induced rat colitis model, AQSA-Glu enhanced the anticolitic potency of 5-AIQ. Furthermore, ABSA was more effective against rat colitis than SSZ or AQSA-Glu, and the anticolitic effects of AQSA-Glu were augmented by combined treatment with a colon-targeted 5-ASA prodrug. In addition, the colon-targeted delivery of PARP inhibitors substantially reduced their systemic absorption. **Conclusions:** Colon-targeted PARP inhibitors may improve the therapeutic and toxicological properties of inhibitors and synergize the anticolitic effects of 5-ASA.

## 1. Introduction

Inflammatory bowel disease (IBD) is a chronic and intractable inflammatory disorder in which pathological lesions primarily appear in the distal intestinal tract. The two major types of IBD are ulcerative colitis and Crohn’s disease, which have different pathological features, such as the location of pathological lesions and the depth of tissue affected by inflammation [1]. Ulcerative colitis and Crohn’s disease are usually characterized by diarrhea, rectal bleeding, abdominal pain, fatigue, and weight loss. In some individuals, IBD is only a mild illness, whereas in others, it is a debilitating condition that can lead to life-threatening complications [2]. Although its exact etiology has not been fully elucidated, recent studies suggest that environmental factors, including food, trigger the initiation of inflammation in the gastrointestinal (GI) tract of individuals genetically predisposed to uncontrolled immune reactions. This leads to tissue destruction and causes further unresolved inflammation, thus perpetuating the inflammatory cycle in the GI tract [3,4,5].

Drug therapies for the treatment of IBD, which remains incurable via pharmacotherapy, aim to induce remission of inflammation and maintain it for as long as possible [1,4]. The recent introduction of biologics, including antitumor necrosis factor, anti-α_4_β_7_ integrin, and anti-interleukin-12 and interleukin-23 agents, has expanded therapeutic options for IBD treatment. However, current anti-IBD drugs, including aminosalicylates, glucocorticoids, immunosuppressants such as JAK inhibitors, and biologics, do not yield satisfactory clinical outcomes in a considerable proportion of patients with IBD owing to drug resistance, limited efficacy, and adverse side effects from long-term use [5,6,7]. Therefore, there is a substantial unmet medical need to develop new anti-IBD drugs.

Poly(ADP-ribose) polymerases (PARPs) are enzymes with several subtypes that catalyze the poly(ADP-ribosyl)ation of acceptor proteins, including PARP itself, using nicotinamide adenine dinucleotide (NAD+) as a substrate to generate ADP-ribose monomers. PARP is auto-PARylated and activated upon recognition of metabolic, chemical, or radiation-induced single-strand DNA breaks, initiating the signaling cascade involved in single-strand DNA break repair [8,9]. Overactivation of PARP has been suggested to cause depletion of cellular NAD+ and ATP stores, leading to necrotic cell death due to the inhibition of glucose oxidation [10,11]. A growing number of cellular proteins that are PARylated by PARP have been identified in various biological contexts, including DNA repair. PARP is now regarded as an important enzyme that functions beyond DNA damage responses and is involved in many aspects of cell signaling [12,13].

Oxidants and free radicals (which cause DNA damage) produced under various pathophysiological conditions are representative activators of PARP. In the pathological context, activation of PARP plays a role in an active and regulated form of cell necrosis, as well as in promoting various proinflammatory signaling pathways, which are intricately interlinked and form a self-amplifying positive feedforward cycle, facilitating the perpetuation of pathophysiological processes, which, in turn, may induce further PARP activation [9,13,14]. These findings provide a solid rationale for the use of PARP as a promising drug target for the development of cytoprotective and anti-inflammatory drugs. In fact, pharmacological or genetic inhibition of PARP elicits beneficial effects in various animal models of inflammation, such as colitis, arthritis, and asthma [10,11,15,16].

PARP inhibitors, including olaparib, rucaparib, niraparib, and talazoparib, are FDA-approved for the treatment of oncological diseases, especially for treating tumors with a DNA repair deficiency, where blocking the PARP pathway further compromises tumor cells, leading to increased cell death [17]. Considering the great potential of PARP as an anti-inflammatory drug target, it is not surprising that PARP inhibitors have been preclinically tested for the treatment of non-oncological diseases, such as respiratory inflammation, neuroinflammation, and sepsis [14,18,19]. Additionally, PARP inhibitors have been investigated as potential anti-inflammatory agents in experimental colitis [20,21,22,23,24]. In all preclinical studies, PARP inhibitors have shown anti-inflammatory and cytoprotective potential.

Colon-targeted drug delivery (CTDD) implies that the orally administered drug is specifically delivered to the large intestine without considerable loss in the stomach and small intestine. CTDD can be accomplished using both pharmaceutical and prodrug approaches [25]. While pharmaceutical approaches utilize techniques such as pH- or time-controlled or pH/enzyme dual-sensitive coating, the prodrug approach involves designing pharmacologically inactive derivatives of the drug whose systemic absorption and pre-systemic metabolism are limited and can be easily converted to the parent drug in the large intestine [25,26]. In general, CTDD tends to increase drug availability in the large intestine and reduce systemic absorption of the drug at the same time [27]. Therefore, this delivery technique can improve the therapeutic potency and safety of drugs intended for the local treatment of colonic diseases such as IBD [28,29,30]. In parallel with its pharmaceutical benefits, CTDD facilitates the repositioning of riluzole and sofalcone as anticolitic drugs by enhancing their safety and therapeutic effectiveness [30,31].

Effective doses of PARP inhibitor tended to be lower than those used in cancer models [32]. Considering that PARP inhibitors, which block DNA repair, are associated with genotoxicity, this difference has clear advantages for therapeutic repurposing in non-oncological diseases, including inflammation [32,33]. However, owing to toxicological concerns, PARP inhibitors are not recommended for the therapeutic repurposing of non-oncological diseases requiring long-term treatment [32]. In this study, we investigated whether CTDD could improve the therapeutic and toxicological properties of PARP inhibitors repositioned as anticolitic drugs and whether PARP inhibitors synergized the anticolitic effects of mesalazine (5-aminosalicylic acid, 5-ASA), an anti-inflammatory drug currently used for the treatment of IBD. For this purpose, we designed and synthesized colon-targeted prodrugs of the PARP inhibitors 3-aminobenzamide (3-AB) and 5-aminoisoquinoline (5-AIQ), which elicit significant PARP inhibition at millimolar and micromolar concentrations, respectively [34,35]. The PARP inhibitors were selected for the following reasons: 1) they have an aniline moiety easily modifiable to a colon-targeted prodrug via azo coupling with the hydrophilic pro-moiety 5-ASA; 2) considering that colonic concentrations of 5-ASA after oral administration of sulfasalazine (SSZ), a colon-targeted anti-IBD prodrug of 5-ASA, at an effective dose reach millimolar levels [29], 5-AIQ is suitable to design a colon-targeted prodrug of the PARP inhibitor acting alone against colitis. Furthermore, 3-AB allows the design of a colon-targeted prodrug to deliver both 5-ASA and 3-AB to the target site at therapeutic (millimolar) concentrations of 5-ASA and 3-AB, thus acting as a mutual prodrug against colitis. The colon specificity of the PARP inhibitor derivatives was evaluated through in vitro and in vivo experiments. In a DNBS-induced rat colitis model, we examined whether colon-targeted PARP inhibitors enhanced the anticolitic activity of PARP inhibitors and synergized the anticolitic effects of 5-ASA. Finally, we examined the ability of CTDD to reduce the risk of systemic side effects associated with PARP inhibitors.

## 2. Materials and Methods

### 2.1. Materials

Salicylic acid (SA), 5-AIQ, 3-AB, sodium nitrite (NaNO_2_), sulfamic acid, and 2,4-dinitrobenzene sulfonic acid (DNBS) were purchased from the Tokyo Chemical Indus-try Co., Ltd. (Tokyo, Japan). Sulfasalazine (SSZ) and aspirin were purchased from Sigma-Aldrich Chemical Co. Inc. (St. Louis, MO, USA). *L*-aspartic acid dimethyl ester (AspDME) hydrochloride, *L*-glutamic acid dimethyl ester (GluDME) hydrochloride, 1, 1′-carbonyldiimidazole (CDI), and olsalazine (OSZ) were purchased from Ambeed, Inc. (Arlington Heights, IL, USA). Reaction solvents and high-performance liquid chromatography (HPLC)-grade solvents were obtained from Junsei Chemical Co. (Tokyo, Japan) and Daejung Chemicals & Metals Co. Ltd. (Gyeonggi-do, Siheung, South Korea), respectively. Cytokine-induced neutrophil chemoattractant-3 (CINC-3) enzyme-linked immunosorbent assay (ELISA) kit was purchased from R&D Systems Inc. (Minneapolis, MN, USA). Phosphate buffer saline (pH 7.4, PBS) was purchased from Thermo Fisher Scientific (Waltham, MA, USA). All other chemicals used were commercially available reagents. Spots on thin-layer chromatography (TLC) plates (silica gel F254s, Merck Millipore, Burlington, MA, USA) were detected using an ultraviolet lamp (254 nm). Infrared (IR) and proton-nuclear magnetic resonance (^1^H-NMR) spectra were taken by a Varian Fourier transform IR spectrophotometer (Varian Medical Systems, Palo Alto, CA, USA) and a Varian AS 500 NMR spectrophotometer (Varian Medical Systems, Palo Alto, CA, USA), respectively.

### 2.2. Synthesis of Derivatives of PARP Inhibitors

To synthesize 5-[(3-carbamoylphenyl)diazenyl]-2-hydroxybenzoic acid (3-AB azo-linked with SA, ABSA), 3-AB (136 mg) and NaNO_2_ (138 mg) were dissolved in 10 mL of pre-chilled 5 M hydrochloric acid (HCl) and stirred at 4 °C for 1 h, followed by the addition of sulfamic acid (98 mg). SA (276 mg) dissolved in 1 M NaOH (10 mL) was added to the reaction solution and reacted at 25 °C for 4 h with maintaining pH 9 by addition of 1 M NaOH or 1 M HCl. The pH of the reaction solution was adjusted to approximately 3. Precipitate formed during pH adjustment was isolated by centrifugation at 4000× *g* for 3 min and washed three times with diethyl ether/acetone (2:1), followed by drying in a vacuum oven. ABSA (M.W.: 285.26); yield: 81%; mp: 280 °C (decomposition); IR (nujol mull), νmax (cm^−1^): 1665 (C=O, -COOH in SA), 1621 (C=O, -CONH in 3-AB); ^1^H-NMR (DMSO-d6): δ 8.37–8.30 (m, 2H), 8.21 (s, 1H), 8.02–7.95 (m, J = 9.6, 7.7 Hz, 3H), 7.64 (t, J = 7.8 Hz, 1H), 7.52 (s, 1H), 7.01 (d, J = 8.8 Hz, 1H). The same synthetic method was used to prepare 2-hydroxy-5-(isoquinolin-5-yldiazenyl)benzoic acid (5-AIQ azo-linked with SA, AQSA). AQSA (M.W.: 293.28); yield: 85%; mp: 298 °C (decomposition); IR (nujol mull), νmax (cm^−1^): 1663 (C=O, -COOH in SA), ^1^H-NMR (DMSO-d6): δ 10.08 (s, 1H), 9.17 (s, J = 5.9 Hz, 1H), 8.82 (d, J = 5.9 Hz, 1H), 8.72 (d, J = 7.8 Hz, 1H), 8.50 (s, 1H), 8.36 (d, J = 7.6 Hz, 1H), 8.32 (d, J = 8.9 Hz, 1H), 8.13 (t, J = 7.3 Hz, 1H), 7.25 (d, J = 8.9 Hz, 1H).

To synthesize *L*-aspartic acid-conjugated AQSA (AQSA-Asp), aspirin (180 mg) dissolved in 20 mL of acetonitrile (ACN) was reacted with CDI (203 mg) at 25 °C for 2 h, followed by the addition of AspDME HCl (198 mg) and triethylamine (0.3 mL). After 24 h, the reaction solvent was evaporated, and the residue dissolved in EA was washed three times with 0.1 M HCl and 5% NaHCO_3_ solution and dehydrated over anhydrous Na_2_SO_4_. After removing the solvent, the residue was reacted with 1 M NaOH (10 mL) at 40 °C for 2 h to yield Asp-conjugated SA (SA-Asp). 5-AIQ (144 mg) and NaNO_2_ (138.0 mg) were dissolved in 5 M HCl (20 mL) and stirred for 1 h at 4 °C, followed by the addition of sulfamic acid (97 mg). SA-Asp dissolved in 1 M NaOH (10 mL) was added dropwise to the reaction solution, which was adjusted to pH 6.0 using 1 M NaOH. Precipitate formed during reaction at 25 °C for 4 h was collected by centrifugation and washed with diethyl ether/acetone (2:1) until no 5-AIQ was detected on TLC. A brown powder was obtained after drying in a vacuum oven. AQSA-Asp (M.W.: 408.3); yield: 68%; mp: 271 °C; IR (nujol mull), ν_max_ (cm^−1^): 1692 and 1646 (-C=O, carboxylic), 1612 cm^−1^ (-C=O, amide); ^1^H-NMR (DMSO-d6): δ 11.49 (s, 1H), 9.36 (s, 1H), 8.61–8.57 (m, 2H), 8.44–8.39 (d, 1H), 8.04 (d, J = 8.8 Hz, 1H), 7.92 (d, J = 7.5 Hz, 1H), 7.83 (dd, J = 9.1, 2.3 Hz, 1H), 7.72 (t, J = 7.8 Hz, 1H), 6.43 (d, J = 9.0 Hz, 1H), 4.46 (d, J = 7.5 Hz, 1H), 2.51 (d, J = 1.8 Hz, 2H). Glutamic acid-conjugated AQSA (AQSA-Glu) was synthesized using GluDME HCl instead of AspDME HCl by the same synthetic method. AQSA-Glu (M.W.: 422.4); yield: 67%; mp: 302 °C; IR (nujol mull), ν_max_ (cm^−1^): 1696 and 1647 (-C=O, carboxylic), 1608 cm^−1^ (-C=O, amide); ^1^H-NMR (DMSO-d6): δ 11.40 (s, 1H), 9.36 (s, 1H), 8.58 (m, J = 5.8 Hz, 2H), 8.46 (d, 1H), 8.06 (d, J = 8.0 Hz, 1H), 7.92 (d, J = 7.4 Hz, 1H), 7.84 (d, J = 9.1 Hz, 1H), 7.73 (t, J = 7.8 Hz, 1H), 6.48 (d, J = 9.0 Hz, 1H), 4.35 (d, 1H), 2.32–2.19 (d, 2H), 2.01–1.84 (d, 2H).

### 2.3. HPLC Analysis

Drugs in the samples were quantitatively analyzed using an HPLC system (Gilson, Middleton, WI, USA) with a Symmetry C_18_ column (Hector, Theale, Berkshire, UK; 250 × 4.6 mm, 5 μm) for chromatographic separation of the drugs. The samples from each experiment were subjected to HPLC after filtration through membrane filters (0.45 μm). The mobile phases A and D consisted of 0.1 M potassium monophosphate solution and ACN (7:3) and 1.0 mM phosphate buffer (pH 7.4) and ACN (8.5: 1.5), containing 0.5 mM tetrabutylammonium chloride. Mobile phases B (8:2) and C (6:4) consisted of distilled water and ACN. The eluate (at a flow rate of 1 mL/min) was monitored at 261 nm (for 5-AIQ), 242 nm (for 3-AB), 386 (for ABSA), 411 nm (for AQSA, AQSA-Asp, and AQSA-Glu), and 330 nm (for 5-ASA) using a UV detector (Gilson, Madison, WI, USA) with a sensitivity of AUFS 0.01. The retention times of 5-AIQ (using mobile phase A), 3-AB, ABSA (using mobile phase B), AQSA, AQSA-Asp, and AQSA-Glu (using mobile phase C) and 5-ASA (using mobile phase D) were 5.6 min, 6.1 min, 4.3 min, 7.1 min, 5.4 min, 5.6 min, and 7.8 min, respectively.

### 2.4. Distribution Coefficient, Chemical Stability, and Cell Permeability

Distribution coefficients (log *D_6.8_*) of the PARP inhibitors and their derivatives were measured using a pH 6.8 isotonic phosphate buffer/n-octanol system, as described in a previous paper [30].

The chemical stability of the derivatives was tested in pH 1.2 HCl-NaCl buffer and pH 6.8 isotonic phosphate buffer. Changes in the concentrations of the derivatives were monitored by HPLC.

Caco-2 cells (4 × 10^5^ per insert) were seeded and grown in Transwell 6-well inserts with a pore size of 0.4 μm (SPL Inc., Houston, TX, USA) until the transepithelial electrical resistance value (EMD Millipore, Billerica, MA, USA) reached 2000 Ω.cm^2^. After removing the culture medium (DMEM) containing 1% penicillin/streptomycin from the basolateral and apical (insert) compartments, the apical compartment was filled with DMEM without phenol red (2 mL) containing PARP inhibitors and their derivatives (500 μM), whereas the basolateral compartment was filled with DMEM without phenol red (3 mL). At appropriate time intervals, the drug concentration in the basolateral compartment was determined by HPLC.

### 2.5. Incubation of Drugs in the Contents of the Small Intestine and the Cecum of Rats

Male Sprague–Dawley rats (250–260 g) were euthanized using CO_2_ gas before a midline incision was made. The contents of the intestinal tract were collected from the small intestine and cecum obtained from each of three rats and were suspended in pH 6.8 isotonic phosphate buffer to prepare a 20% (*w*/*v*) suspension. To maintain anaerobic conditions in the cecum, the cecal contents were collected under a N_2_ atmosphere in an atmospheric bag (AtmosBag, Sigma, St. Louis, MO, USA). Derivatives of PARP inhibitors (2 mM) dissolved in pH 6.8 isotonic phosphate buffer (5 mL) were mixed with the cecal and small intestinal suspensions (5 mL) and then incubated at 37 °C. The cecal contents were incubated in nitrogen. At predetermined time intervals, a 0.5 mL portion of the mixture was transferred to a microtube and centrifuged at 10,000× *g* at 4 °C for 10 min. The supernatants (0.1 mL) were mixed with MeOH (0.9 mL), followed by centrifugation at 10,000× *g* at 4 °C for 7 min. The supernatants were filtered through a membrane filter (0.45 μm), and the concentrations of PARP inhibitors in the filtrates (20 μL) were analyzed using HPLC.

### 2.6. Analysis of Drug Concentration in Blood and Cecum

Male Sprague–Dawley rats were fasted for 24 h but had access to water. 5-AIQ (10 mg/kg), AQSA-Glu (equivalent to 10 mg/kg 5-AIQ), 3-AB (17 mg/kg), and ABSA (equivalent to 17 mg/kg 3-AB) in PBS (1.0 mL) were administered to the rats by oral gavage. Blood samples were collected via cardiac puncture at 2, 4, and 8 h after the oral gavage. The blood samples were centrifuged at 10,000× *g* at 4 °C for 10 min to obtain the plasma (0.3 mL). The plasma (0.1 mL) was transferred to a centrifuge tube and mixed with methanol (0.9 mL). The supernatants were filtered through a membrane filter (0.45 μm), and then the filtrate (20.0 μL) was subjected to HPLC analysis. To determine drug concentrations in the cecum, cecal contents were collected at 2, 4, and 8 h after oral gavage and mixed with pH 6.8 isotonic phosphate buffer to make a 10% suspension, which was subjected to centrifugation at 10,000× *g* for 10 min at 4 °C. The supernatants (0.1 mL) were transferred to fresh microtubes, followed by the addition of MeOH (0.9 mL). After centrifugation at 10,000× *g* at 4 °C for 7 min and subsequent filtration through a membrane filter (0.45 μm), the concentrations of PARP inhibitors in the filtrates (20 μL) were analyzed using HPLC.

### 2.7. Evaluation of Anticolitic Effects

DNBS-induced colitis was produced in rats as previously described [29]. Two independent animal experiments were performed to evaluate the anticolitic effects of the drugs. For the first experiment, the rats were divided into the following five treatment groups (n = 5 per group): group 1 (normal group), oral gavage of 1.0 mL of PBS; group 2 (colitis group), oral gavage of 1.0 mL of PBS; group 3 (5-AIQ-treated colitis group), oral gavage of 5-AIQ (5 mg/kg) in 1.0 mL of PBS; group 4 [AQSA-Glu-treated colitis group (L)], oral gavage of AQSA-Glu (7.5 mg/kg, equivalent to 2.5 mg/kg of 5-AIQ) in 1.0 mL of PBS; and group 5 [AQSA-Glu-treated colitis group (H)], oral gavage of AQSA-Glu (15 mg/kg, equivalent to 5 mg/kg of 5-AIQ) in 1.0 mL of PBS. For the second experiment, the rats were divided into the following six treatment groups (n = 5 per group): group 1 (normal group), oral gavage of 1.0 mL of PBS; group 2 (colitis group), oral gavage of 1.0 mL of PBS; group 3 (SSZ-treated colitis group), oral gavage of SSZ (50 mg/kg) in 1.0 mL of PBS; group 4 (AQSA-Glu-treated colitis group), oral gavage of AQSA-Glu (15 mg/kg) in 1.0 mL of PBS; group 5 (mixture of AQSA-Glu + OSZ-treated colitis group), oral gavage of mixture of AQSA-Glu (15 mg/kg) and OSZ (19 mg/kg, half-equimolar to 50 mg/kg of SSZ) in 1.0 mL of PBS; and group 6 (ABSA-treated colitis group), oral gavage of ABSA (36 mg/kg, equivalent to 50 mg/kg of SSZ) in 1.0 mL of PBS. Three days after the induction of inflammation, each drug was orally administered to rats once daily, and the anticolitic effects were assessed 24 h after the sixth medication. The animal experiment scheme is shown in Supplementary Data S1. The colonic damage score was calculated using a scoring system modified from previously reported criteria. The modified scoring system is shown in Supplementary Data S2. Four independent observers who were blinded to the treatment conditions scored colonic damage according to the scoring system. Myeloperoxidase (MPO) activity in the distal colon (4 cm) was measured as described previously [29].

### 2.8. Western Blot Analysis and ELISA

Tissue samples (0.2 g/mL) were homogenized in pre-chilled radioimmunoprecipitation assay buffer (50 mM Tris-HCl [pH 7.4], 1 mM EDTA, 0.7% Na deoxycholate, 1% NP-40, 150 mM NaCl, 0.3 μM aprotinin, 1 μM pepstatin, and 1 mM phenylmethylsulfonyl fluoride). The homogenates were centrifuged at 10,000× *g* at 4 °C for 10 min, followed by agitation on ice for 30 min. Whole and nuclear cell lysates were prepared as described previously [31]. The protein concentrations in the lysates were determined using bicinchoninic acid (Thermo Fisher Scientific, Waltham, MA, USA) according to the manufacturer’s instructions.

Cell and tissue lysates were separated by SDS-PAGE on 10% and 7.5% gels. Nrf2, HO-1, cyclooxygenase-2 (COX-2), and inducible nitric oxide synthase (iNOS) proteins were detected using the following antibodies: anti-Nrf2 (sc-365949, Santa Cruz Biotechnology, Dallas, TX, USA), anti-HO-1 (sc-136961, Santa Cruz Biotechnology), anti-COX-2 (sc-365374, Santa Cruz Biotechnology), anti-iNOS (NOS-2) antibody (sc-7271, Santa Cruz Biotechnology), and secondary antibodies corresponding to the primary antibodies (Santa Cruz Biotechnology). Protein bands were visualized using SuperSignal chemiluminescence substrate (Thermo Fisher Scientific). α-Tubulin were detected using antibodies specific to each protein (Santa Cruz Biotechnology) as loading controls. Western blot images were quantified using Image Lab software (version 5.2 build 14; Bio-Rad, Hercules, CA, USA). The means of the quantified values are presented under each Western blot (n = 3 for cell experiments and n = 5 for animal experiments).

The levels of the inflammatory cytokine-induced neutrophil chemoattractant-3 (CINC-3) in the inflamed distal colon were determined using a CINC-3 ELISA kit (R&D Systems, Minneapolis, MN, USA). Distal colon samples were minced in potassium phosphate buffer (pH 6.0), homogenized, and centrifuged at 10,000× *g* at 4 °C for 10 min. The supernatants were subjected to ELISA according to the manufacturer’s instructions.

### 2.9. Data Analysis

The results are expressed as the mean ± standard deviation. One-way analysis of variance followed by Tukey’s Honestly Significant Difference test and the Mann–Whitney *U* test (used only for colonic damage score) were employed to test the differences between groups. Differences were considered statistically significant at *p* < 0.05.

## 3. Results

### 3.1. Synthesis of Derivatives of PARP Inhibitors

**Figure 1:**
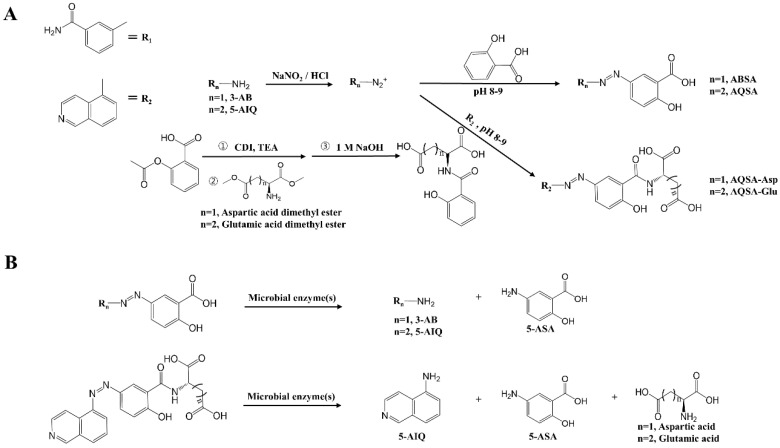
**Synthetic scheme and colonic activation of derivatives of PARP inhibitors.** (**A**) Synthetic scheme of derivatives of PARP inhibitors. 3-AB: 3-aminobenzamide, 5-AIQ: 5-aminoisoquinoline, ABSA: 3-AB azo-linked with SA, AQSA: 5-AIQ azo-linked with SA, ACN: acetonitrile, CDI: 1,1′-carbonydiimidazole, AQSA-Asp: *L*-aspartic acid-conjugated AQSA, AQSA-Glu: *L*-glutamic acid-conjugated AQSA. (**B**) Colonic activation of derivatives of PARP inhibitors. 5-ASA: 5-aminosalicylic acid.

To impose colon specificity on the PARP inhibitors, 5-AIQ and 3-AB were azo-linked with SA via an azo-coupling reaction, yielding AQSA and ABSA. The reaction proceeded easily and in good yield. Because 5-AIQ (log *D_6.8_*: 3.16) is more lipophilic than 3-AB (log *D_6.8_*: 0.33), it is unlikely that conjugation with SA confers sufficient hydrophilicity to substantially decrease the passive transport of 5-AIQ through the epithelial cell layer in the GI tract. Therefore, further modifications to increase hydrophilicity were implemented to enhance its ability to prevent passive transport via the cell membrane. AQSA was conjugated with the acidic amino acids aspartic acid (Asp) and glutamic acid (Glu) to produce AQSA-Asp and AQSA-Glu. The synthetic methods for the derivatives of PARP inhibitors and the proposed scheme for colonic activation of the derivatives are shown in Figure 1A,B. The formation of the derivatives was verified by FTIR and ^1^H-NMR. In the FTIR spectra (Supplementary Data S3A), the PARP inhibitors azo-coupled with SA, AQSA, and ABSA showed a carbonyl band derived from SA, whereas broad carbonyl bands (ascribed to the two carboxylic acid groups in acidic amino acids and the amide bond formed by the amide conjugation of SA with acidic amino acids) were observed in the amino acid-conjugated AQSA. In the ^1^H-NMR spectra (Supplementary Data S3B), the aromatic proton signals in the derivatives were downfield-shifted by azo coupling, and aliphatic proton signals ascribed to acidic amino acids were observed in the amino acid-conjugated AQSA.

### 3.2. Colon Specificity of Derivatives of PARP Inhibitors

**Figure 2:**
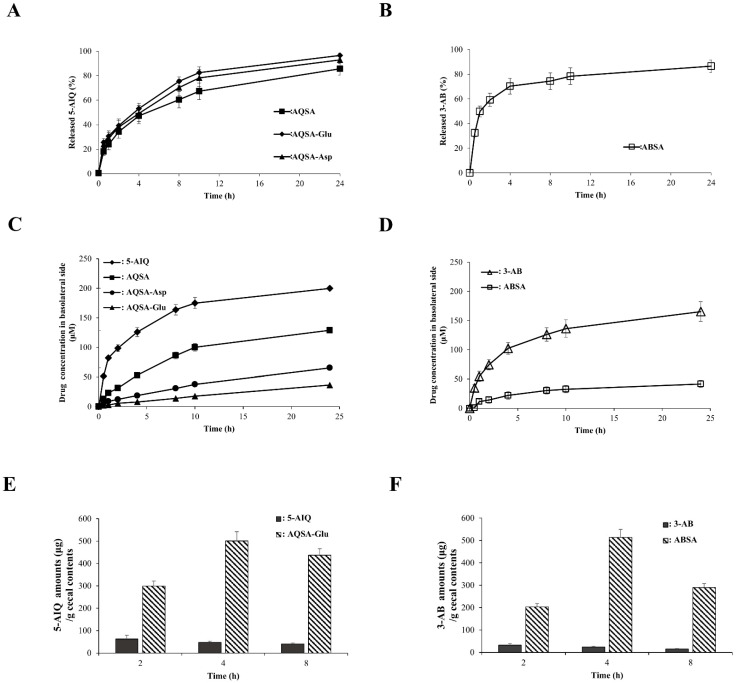
**Derivatives of PARP inhibitors are colon-specific.** (**A**) AQSA, AQSA-Glu, and AQSA-Asp (1 mM) were incubated with 10% cecal contents suspended in PBS (pH 6.8) under nitrogen. The concentrations of 5-AIQ were analyzed using HPLC at appropriate time intervals. (**B**) The same experiment was conducted using ABSA (1 mM). (**C**) 5-AIQ, AQSA, AQSA-Glu, and AQSA-Asp (500 µM, 2 mL) dissolved in DMEM medium without phenol red were added to the apical compartment of the Caco-2 cell monolayer. At appropriate time intervals, the concentrations of each drug were determined in the basolateral compartment filled with the medium (3 mL) using HPLC. (**D**) The same experiment was conducted using 3-AB and ABSA (500 µM, 2 mL). (**E**,**F**) 5-AIQ (10.0 mg/kg) or AQSA-Glu (29.3 mg/kg, equivalent to 10 mg/kg of 5-AIQ) suspended in PBS (1 mL) was administered orally to rats. The rats were killed 2, 4, and 8 h after oral administration (**E**). The same experiment was conducted using 3-AB (17 mg/kg) and ABSA (36 mg/kg, equivalent to 17 mg/kg of 3-AB) (**F**). The concentrations of 5-AIQ and 3-AB in the cecum were analyzed using HPLC. The data in (**A**–**F**) are presented as mean ± SD (n = 3).

To assess the colon specificity of the derivatives of PARP inhibitors, we first tested whether the derivatives remained intact during transit through the stomach and small intestine. The derivatives were incubated in buffers of pH 1.2 and 6.8, representing the luminal pH of the stomach and small intestine, and in the small intestinal contents of rats. No significant changes in the concentrations of the derivatives were observed during incubation for up to 10 h. Subsequently, we tested whether, upon arrival in the large intestine, the derivatives were activated to their parent drugs, 5-AIQ and 3-AB. The derivatives were incubated with the cecal contents of rats. To simulate the anaerobic state in the large intestine, this experiment was performed in a glove bag in which air was replaced with nitrogen. As shown in Figure 2A,B, all the derivatives were converted to the corresponding parent drugs, 5-AIQ and 3-AB. The conversion percentages of the derivatives were similar at 24 h, although the conversion rate of ABSA was slightly higher than that of the derivatives of 5-AIQ. We also tested whether the derivatives traveled to the large intestine without substantial loss owing to systemic absorption in the upper intestine. The distribution coefficient (log *D_6.8_*), a parameter that predicts systemic absorption via passive transport in the GI tract, was measured using a 1-octanol/isotonic phosphate buffer (pH 6.8) system. Conjugation of the PARP inhibitors with SA lowered the log *D_6.8_* of 5-AIQ (3.16) and 3-AB (0.33) to 0.31 (for AQSA) and −1.07 (for ABSA), respectively. Additional conjugation of AQSA with acidic amino acids further reduced the log *D_6.8_* of AQSA to −1.77 (for AQSA-Asp) and −1.83 (for AQSA-Glu). To further test their passive transport ability through the epithelial cell layer, the derivatives were subjected to cell permeability testing using a Caco-2 cell monolayer. The concentrations of the derivatives and PARP inhibitors were determined on the acceptor (basolateral) side at appropriate time intervals after adding the drugs to the donor (apical) side. As shown in Figure 2C,D, the transport of the derivatives via the Caco-2 cell monolayer was retarded compared with that of the corresponding PARP inhibitors. Additional conjugation with acidic amino acids further retarded AQSA transport. Overall, the transport rates correlated with the log *D_6.8_* of the tested compounds. These results suggest that the colonic delivery efficiency of PARP inhibitors and their derivatives followed the order AQSA-Glu ≈ AQSA-Asp > AQSA > 5-AIQ and ABSA > 3-AB, and all derivatives were effectively activated into the parent drugs (PARP inhibitors) after arrival in the large intestine. To validate the in vitro assessment of colon specificity, equimolar doses of 5-AIQ and AQSA-Glu were orally administered to rats, and concentrations of 5-AIQ were determined in the cecum 2, 4, and 8 h after oral administration. The same experiment was performed using 3-AB and ABSA. As shown in Figure 2E,F, in parallel with the in vitro results, oral administration of AQSA-Glu and ABSA delivered a much greater amount of the corresponding PARP inhibitors to the cecum than that of the PARP inhibitors, and peak concentrations of the PARP inhibitors were observed at 4 h. These results indicated that the derivatives of PARP inhibitors are colon-specific, thus acting as colon-targeted prodrugs of PARP inhibitors.

### 3.3. Colon-Targeted Delivery of 5-AIQ Enhances Its Anticolitic Activity

**Figure 3:**
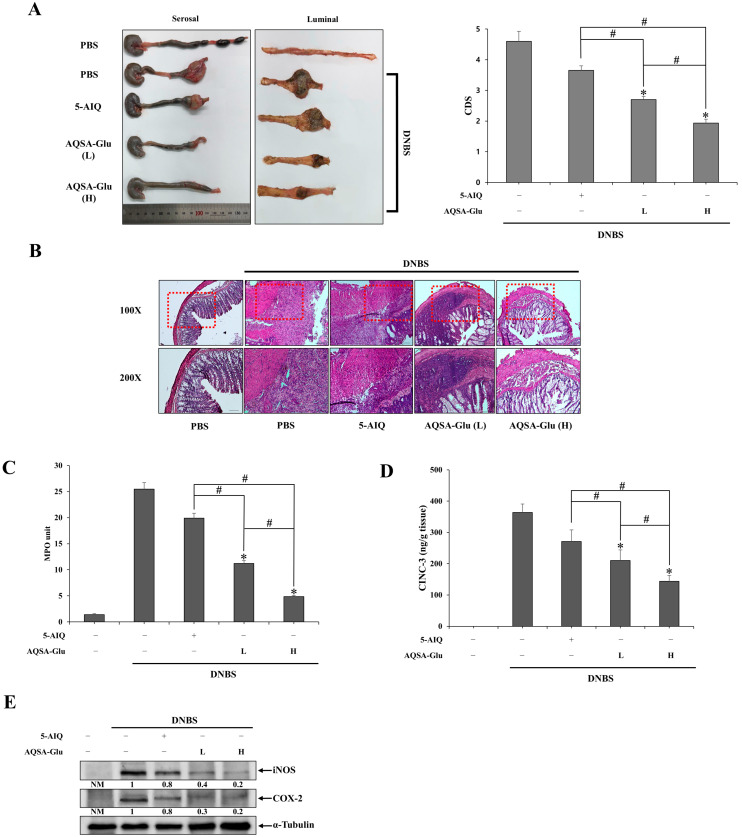
**AQSA-Glu potentiates the anticolitic activity of 5-AIQ.** Three days after colitis induction by DNBS, 5-AIQ (5 mg/kg) and AQSA-Glu [7.5 mg/kg, equivalent to 2.5 mg/kg of 5-AIQ (L) and 15 mg/kg, equivalent to 5 mg/kg of 5-AIQ (H)] were administered orally to rats once per day, and the rats were euthanized 24 h after the sixth treatment. (**A**) Left panel: photos of the distal colons of rats in which serosal and luminal sides are shown separately. Right panel: overall colonic damage was scored for each group and presented as colonic damage score (CDS). * α < 0.05 vs. DNBS control. (**B**) H & E staining was performed with the colonic tissue sections of rats subjected to various treatments. Upper panel: representative images of 100× magnification. Lower panel: representative images of 200× magnification for the dotted boxes in the upper panel. In the inflamed distal colons (4.0 cm), (**C**) myeloperoxidase (MPO) activity was measured in addition to determining the levels of (**D**) CINC-3 and (**E**) iNOS and COX-2 using an Elisa kit and Western blotting. A loading control (α-Tubulin) was used for Western blot analysis of COX-2 and iNOS. NM: not measurable. The data are represented as mean ± SD (n = 5). * *p* < 0.05 vs. DNBS control ^#^ *p* < 0.05.

We examined whether a colon-targeted PARP inhibitor elicited anticolitic effects in a rat model of colitis and enhanced the anticolitic activity of the PARP inhibitor. For this experiment, AQSA-Glu was chosen because of its high colon-specific performance and lack of 5-ASA release upon colonic activation, thus allowing for a clear interpretation of the therapeutic effects of colon-targeted 5-AIQ against colitis. As reported previously [36], no 5-ASA was detected in the cecal contents upon incubation with AQSA-Glu or in the cecum after oral administration of AQSA-Glu. 5-AIQ (5 mg/kg) and AQSA-Glu (equivalent to 2.5 and 5 mg/kg of 5-AIQ) were orally administered to colitic rats induced by DNBS once per day for 6 days, and the anticolitic effects of the drugs were monitored. As shown in Figure 3A,B, DNBS severely damaged the distal colon of rats, resulting in extensive mucosal defoliation, mucosal ulcers covered with hemorrhagic scabs, tissue edema, stricture, shortening of the colon, and adhesion to neighboring organs. Both doses of AQSA-Glu substantially improved colonic damage in a dose-dependent manner and were more effective in ameliorating colonic damage than 5-AIQ. H&E staining of the inflamed distal colon showed greater mucosal recovery with AQSA-Glu. In addition, AQSA-Glu was more effective in alleviating colonic inflammation than 5-AIQ. As shown in Figure 3C–E, the levels of MPO activity (Figure 3C) and proinflammatory mediators CINC-3 (Figure 3D), iNOS, and COX-2 (Figure 3E), which were elevated by colonic inflammation, were decreased, and AQSA-Glu at both low and high doses was more effective in suppressing MPO activity and reducing proinflammatory mediators in the inflamed distal colon than 5-AIQ, clearly indicating that colon-targeted delivery of 5-AIQ enhanced the therapeutic potency of 5-AIQ against rat colitis.

### 3.4. PARP Inhibitors Synergize the Anticolitic Effects of 5-ASA

**Figure 4:**
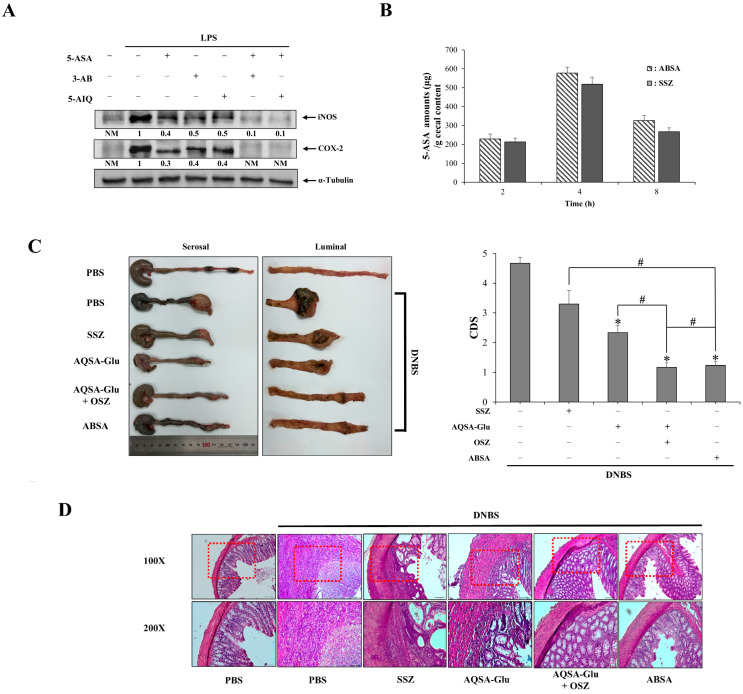

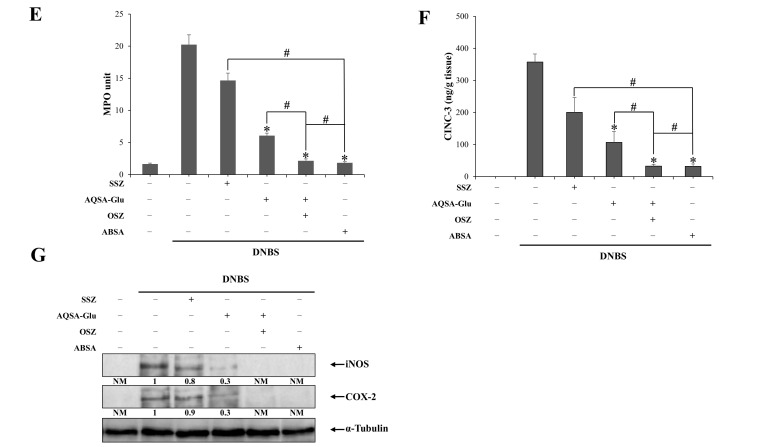
**Colon-targeted PARP inhibitors synergize the anticolitic effects of mesalazine.** (**A**) RAW264.7 cells pretreated with 5-ASA (20 mM), 3-AB (1 mM), and 5-AIQ (10 μM) for 1 h were challenged with LPS for 24 h. The levels of iNOS and COX-2 proteins were analyzed using Western blotting. (**B**) SSZ (50 mg/kg) and ABSA (36 mg/kg, equimolar to 50 mg/kg of SSZ) suspended in PBS (1 mL) were administered orally to rats. The rats were killed 2, 4, and 8 h after oral administration. The concentrations of 5-ASA in the cecum were analyzed using HPLC. (**C**) Three days after colitis induction by DNBS, SSZ (50 mg/kg), AQSA-Glu (15 mg/kg), a mixture of AQSA-Glu (15 mg/kg) + olsalazine (OSZ, 19 mg/kg, half-equimolar to 50 mg/kg of SSZ), and ABSA (36 mg/kg, equimolar to 50 mg/kg of SSZ) were administered orally to rats once per day, and the rats were euthanized 24 h after the sixth treatment. (**C**) Left panel: photos of the distal colons of rats where serosal and luminal sides are shown separately. Right panel: overall colonic damage was scored for each group and presented as colonic damage score (CDS). * α < 0.05 vs. DNBS control. (**D**) H & E staining was performed with the colonic tissue sections of rats subjected to various treatments. Upper panel: representative images of 100× magnification. Lower panel: representative images of 200× magnification for the dotted boxes in the upper panel. In the inflamed distal colons (4.0 cm), (**E**) myeloperoxidase (MPO) activity was measured in addition to determining the levels of (**F**) CINC-3 and (**G**) iNOS and COX-2 using an Elisa kit and Western blotting. A loading control (α-Tubulin) was used for Western blot analysis of COX-2 and iNOS. NM: not measurable. The data are represented as mean ± SD (n = 5). * *p* < 0.05 vs. DNBS control ^#^ *p* < 0.05.

We examined whether colon-targeted PARP inhibitors could cooperate therapeutically with 5-ASA for the treatment of rat colitis, thus acting as a mutual prodrug with improved anticolitic efficacy. To test this possibility in cells, murine macrophage RAW264.7 cells challenged with LPS were treated with each PARP inhibitor and/or 5-ASA, both of which are known to inhibit NF-κB activity [37,38], which plays a critical role in inflammation and immune response [39]. The LPS-mediated induction of the proinflammatory mediators iNOS and COX-2 was then monitored. As shown in Figure 4A, single treatment with each PARP inhibitor or 5-ASA reduced the levels of iNOS and COX-2 proteins, and combined treatment with each PARP inhibitor and 5-ASA suppressed the induction of proinflammatory mediators in an additive manner, supporting the idea that a PARP inhibitor could synergize the anti-inflammatory effects of 5-ASA. We examined the combined therapeutic effects of 5-ASA and a PARP inhibitor in a rat model of DNBS-induced colitis. Unlike the prodrug design of 5-AIQ, the prodrug design of 3-AB aimed at liberating both the PARP inhibitor 3-AB and the current anti-IBD drug 5-ASA at a sufficient concentration to elicit a therapeutic effect against rat colitis. To test whether ABSA was suitable for this therapeutic purpose, cecal accumulation of 5-ASA was compared at 2, 4, and 8 h after the oral administration of ABSA and SSZ, a colon-targeted prodrug of 5-ASA used for the treatment of IBD, at an equimolar dose. As shown in Figure 4B, the amount and temporal pattern of 5-ASA accumulation in the cecum were comparable, indicating that ABSA delivered as much 5-ASA as SSZ to the target site at an effective dose.

Colitic rats were treated orally with ABSA (at an equimolar dose to SSZ), AQSA-Glu (equivalent to 5 mg/kg of 5-AIQ) as a colon-targeted PARP inhibitor, and SSZ (50 mg/kg) as a colon-targeted 5-ASA. In addition, a mixture of AQSA-Glu (equivalent to 5 mg/kg of 5-AIQ) and the colon-targeted azo-homodimer of 5-ASA, OSZ, at a half-equimolar dose to SSZ was included as a treatment group to further validate the combined action of 5-ASA and the PARP inhibitor. DNBS induces severe colitis in rats, accompanied by colonic damage and inflammation. All the drugs ameliorated colonic damage and accelerated mucosal recovery, as shown in Figure 4C,D. ABSA showed greater effectiveness in healing colonic injury, including mucosa, than the other single-treatment groups and was as effective against colonic damage as the mixture. Consistently, as shown in Figure 4E–G, all the drugs lowered the inflammatory indices: MPO activity (Figure 4E) and the levels of proinflammatory mediators CINC-3 (Figure 4F), iNOS, and COX-2 (Figure 4G), which were elevated in the inflamed colon. The anti-inflammatory effectiveness was greater in the treatment groups (ABSA and the mixture), where the combined action of 5-ASA and PARP inhibitors occurred, compared to the single-treatment groups. These results indicated that colon-targeted co-delivery of 5-ASA and PARP inhibitors exerted a synergistic anticolitic action, and ABSA acted as a mutual prodrug for the treatment of rat colitis.

### 3.5. Colon-Targeted Delivery Reduces the Risk of Systemic Side Effects of a PARP Inhibitor

**Figure 5:**
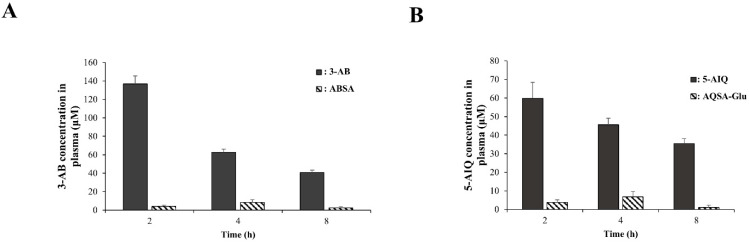
**Colon-targeted PARP inhibitors reduce the risk of systemic side effects of PARP inhibitors.** (**A**) ABSA (36 mg/kg, equivalent to 17 mg/kg of 3-AB) and 3-AB (17 mg/kg) suspended in PBS (1 mL) were administered orally to rats. The rats were killed 2, 4, and 8 h after oral administration. The concentrations of 3-AB in the blood were analyzed using HPLC. (**B**) The same experiment was conducted with 5-AIQ (10 mg/kg) and AQSA-Glu (30 mg/kg, equivalent to 10 mg/kg of 5-AIQ). The data in A and B are presented as mean ± SD (n = 3).

PARP inhibition is a promising therapeutic modality for inflammatory disorders. However, pharmacotherapy for non-oncological diseases using PARP inhibitors is associated with toxicological concerns [32]. Therefore, safety is one of the most important issues to be addressed in the drug repositioning of PARP inhibitors for the treatment of IBD, where long-term pharmacotherapy may be required. Colon-targeted delivery of a drug can reduce systemic side effects by limiting systemic absorption [25]. We examined whether the colon-targeted delivery of PARP inhibitors decreases their systemic absorption, thereby reducing the risk of side effects. ABSA (equivalent to 17 mg/kg of 3-AB) and 3-AB (17 mg/kg) were orally administered to rats, and the concentrations of 3-AB in the blood were determined at 2, 4, and 8 h after oral administration. The same experiment was conducted using AQSA-Glu (equivalent to 10 mg/kg of 5-AIQ) and 5-AIQ (10 mg/kg). As shown in Figure 5A,B, oral administration of the PARP inhibitors resulted in blood concentrations of up to approximately 137 μM (for 3-AB) and 60 μM (for 5-AIQ) at 2 h. Colon-targeted delivery of the PARP inhibitors substantially reduced the maximal blood concentrations of 3-AB and 5-AIQ to 8 μM (for 3-AB) and 7 μM (for 5-AIQ), respectively, which corresponds to 1/17 (for 3-AB) and 1/9 (for 5-AIQ) of the maximal blood concentrations obtained after oral administration of 5-AIQ and 3-AB. These results suggest that colon-targeted delivery of PARP inhibitors reduces toxicological concerns when repositioning PARP inhibitors as anti-IBD drugs.

## 4. Discussion

PARP inhibitors have therapeutic potential in the treatment of diverse non-oncological disorders, including inflammation. Here, we investigated the therapeutic and toxicological benefits of colon-targeted PARP inhibitors in a rat model of IBD. Our results clearly showed that colon-targeted PARP inhibitors improved both the safety and anticolitic potency of PARP inhibitors and synergized the anticolitic effects of 5-ASA.

The PARP inhibitors 5-AIQ and 3-AB were chemically modified to form colon-targeted prodrugs. SA was azo-conjugated to the PARP inhibitors through an aniline moiety, which is chemically available for azo coupling. For 5-AIQ (log *D_6.8_*: 3.16), which is more lipophilic than 3-AB (log *D_6.8_*: 0.33), AQSA underwent additional amide conjugation of the carboxylic group in AQSA with the amino groups in acidic amino acids, likely increasing the hydrophilicity of AQSA. As intended, the log *D_6.8_* of the PARP inhibitors was lowered by simple azo-coupling with SA, and the log *D_6.8_* of AQSA was further lowered by additional conjugation with amino acids. Lowering the log *D_6.8_* of the PARP inhibitors led to retardation of passive transport via the Caco-2 cell monolayer, and the ability of the derivatives to retard passive transport depended on their log *D_6.8_*. In fact, the cell permeability of amino acid-conjugated AQSA, with a lower log *D_6.8_*, was less than that of 5-AIQ and AQSA. ABSA was consistently less cell-permeable than 3-AB. All derivatives released the corresponding PARP inhibitors into the cecal contents while remaining stable in the small intestinal contents and in pH 1.2 buffer. In line with the broad substrate specificity of azo-reductase in the gut microflora [40], the conversion percentages of the derivatives (to PARP inhibitors) were not significantly different.

These in vitro results suggest reduced systemic absorption in the GI tract. Colonic activation of the derivatives was verified by in vivo experiments to evaluate colon specificity. The oral administration of ABSA and AQSA-Glu delivered and accumulated much greater amounts of the corresponding PARP inhibitors in the cecum than that of each PARP inhibitor alone.

Consistent with the known therapeutic benefits of colon-targeted delivery of anti-IBD drugs [25], the colon-targeted delivery of 5-AIQ enhanced the therapeutic potency of 5-AIQ against rat colitis. This was supported by our data, which showed that AQSA-Glu, even at a low dose (2.5 mg/kg), was more effective against rat colitis than 5-AIQ (5 mg/kg). The therapeutic benefit of colon-targeted 5-AIQ was likely due to increased therapeutic availability at the inflamed site (colon), as shown in Figure 2B and Figure 5A, demonstrating that the cecal concentrations obtained after oral AQSA-Glu were much higher than the cecal and blood concentrations obtained after oral administration of 5-AIQ. Furthermore, PARP inhibitors cooperate with 5-ASA to ameliorate colonic damage and inflammation, thereby synergizing with the anticolitic effects of 5-ASA. This was clearly demonstrated by the data showing that ABSA, which delivers and releases both 5-ASA and 3-AB, was more effective against rat colitis than SSZ and AQSA-Glu. In addition, the anticolitic effects of AQSA-Glu were comparable to those of ABSA when co-administered with OSZ (AQSA-Glu + OSZ). For the combined treatment of 5-AIQ with 5-ASA, OSZ was thought to be more suitable than SSZ for a clear interpretation of the combined anticolitic effects, given that while SSZ is activated to sulfapyridine as well as 5-ASA, OSZ is a colon-targeted prodrug activated to two molecules of 5-ASA, thus eliminating concerns about other drug interactions. The cooperative anticolitic effects were at least partly attributed to the additive inhibition of NFκB, a transcription factor that plays a critical role in immune response and inflammation [39], through combined treatment with PARP inhibitors and 5-ASA. This argument was supported by cell experiments showing that combined treatments with 5-ASA and PARP inhibitors were more effective in suppressing the induction of NFκB target gene products, COX-2 and iNOS, in murine macrophages challenged with LPS, than single treatments with either drug. Similarly, the levels of COX-2 and iNOS in the inflamed colon were suppressed to a greater extent by ABSA than by SSZ or AQSA. The inhibitory effects of PARP inhibitors and 5-ASA on NFκB activity are consistent with previous papers [37,38,41,42].

In addition to their enhanced anticolitic activity, colon-targeted PARP inhibitors are likely to improve the safety of PARP inhibitors repositioned as anticolitic drugs. The enhanced anticolitic potency of colon-targeted PARP inhibitors lowers the effective anticolitic dose. Moreover, colon-targeted delivery of PARP inhibitors substantially decreased their blood concentrations, likely limiting systemic absorption. Considering that the toxicity of PARP inhibitors is dose-sensitive and is associated with the blood concentrations of PARP inhibitors [32], the delivery technique is a solid strategy to reduce the risk of side effects and facilitate the repositioning of PARP inhibitors as anticolitic drugs. However, local side effects by colon-targeted PARP inhibitors still need to be considered. This concern may be circumvented by elaborating dosage regimens and finding sensitive biomarkers for monitoring therapy response and long-term effects of colon-targeted PARP inhibitors.

To deliver PARP inhibitors to the target site, colon-targeted prodrugs of PARP inhibitor were designed and synthesized. In general, the prodrug approach has advantages over pharmaceutical formulations such as pH- or time-controlled coating in that the former elicits better colon-specific performance than the latter [25]. Unlike pharmaceutical formulations applicable to most of the candidate drugs for colonic delivery, the prodrug approach requires a functional group of a candidate drug modifiable to a colon-targeted prodrug, thus limiting application of this approach to drugs with the proper structure [25]. Recently, pharmaceutical formulation using pH/enzyme dual-sensitive coating, namely, OPTICORE technology, was applied to colonic delivery of 5-ASA [26]. Given that the coating technique shows improved colon-specific performance compared to traditional coating techniques, it would be interesting to compare colon-specific performance of the OPTICORE technology with the prodrug approach.

Our data showing that colon-targeted PARP inhibitors synergized the anticolitic effects of 5-ASA suggest that the therapeutic limitation of 5-ASA, applicable only to mild to moderate IBD due to its low efficacy [43], can be circumvented by colon-targeted co-delivery of the two drugs, thereby extending the anti-IBD spectrum of 5-ASA to severe IBD. In addition, an azo bond, adopted as a colon-specific link in this study, is a clinically proven chemical link utilized for current anti-IBD colon-targeted prodrugs of 5-ASA, such as SSZ and OSZ [44].

This study provides compelling evidence on therapeutic potential of colon-targeted PARP inhibitors as an anti-IBD drug and a therapeutic partner of 5-ASA and a therapeutic rationale for combination therapy of the FDA-approved PARP inhibitors such as olaparib and rucaparib and the colon-targeted prodrugs of 5-ASA olsalazine and sulfasalazine. A limitation of this study is that long-term local and systemic effects of colon-targeted PARP inhibitors were not investigated, which is required for clarifying toxicological issues concerning the non-oncological use of PARP inhibitors.

## 5. Conclusions

In conclusion, colon-targeted delivery of PARP inhibitors may be a promising strategy to enhance the anticolitic potency and reduce the risk of systemic side effects of PARP inhibitors and augment the anticolitic efficacy of 5-ASA via synergistic action.

## Data Availability

The data presented in this study are available in the article and Supplementary Materials.

## Supplementary Materials

The following supporting information can be downloaded at: https://www.mdpi.com/article/10.3390/pharmaceutics16121546/s1, Data S1: Animal experiment scheme. Data S2: Modified scoring system. Data S3: Instrumental characterization of derivatives of PARP inhibitors: (A) FT-IR spectra, (B) ^1^H-NMR spectra.
