# Supplementary material for: Colon-Targeted Poly(ADP-ribose) Polymerase Inhibitors Synergize Therapeutic Effects of Mesalazine Against Rat Colitis Induced by 2,4-Dinitrobenzenesulfonic Acid
Source: Pharmaceutics. 2024 Dec 2;16(12):1546. doi: 10.3390/pharmaceutics16121546 (PMC11728683; doi:10.3390/pharmaceutics16121546)

Supplementary Data 1. Animal experiment scheme.

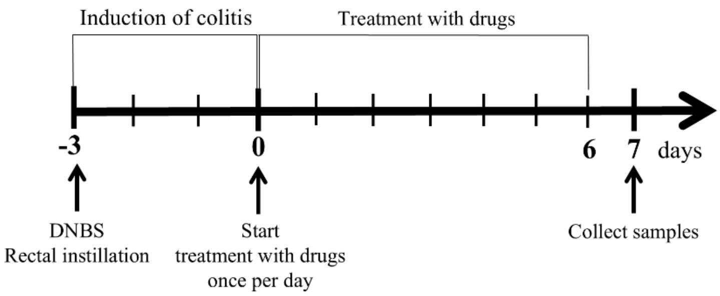

Supplementary Data 2. Modified scoring system

Score	Feature
0	normal appearance
1	localized hyperemia but no ulcer
2	linear ulcers without significant inflammation
3	2–4 cm site of inflammation and ulceration
4	serosal adhesion to other organs, 2–4 cm site of inflammation and ulceration
5	stricture, serosal adhesion involving several bowel loops, <4 cm site of inflammation and ulceration

Supplementary Data 3. Instrumental characterization of derivatives of PARP inhibitors

(A) FT-IR spectra, (B) ^{1}H-NMR spectra

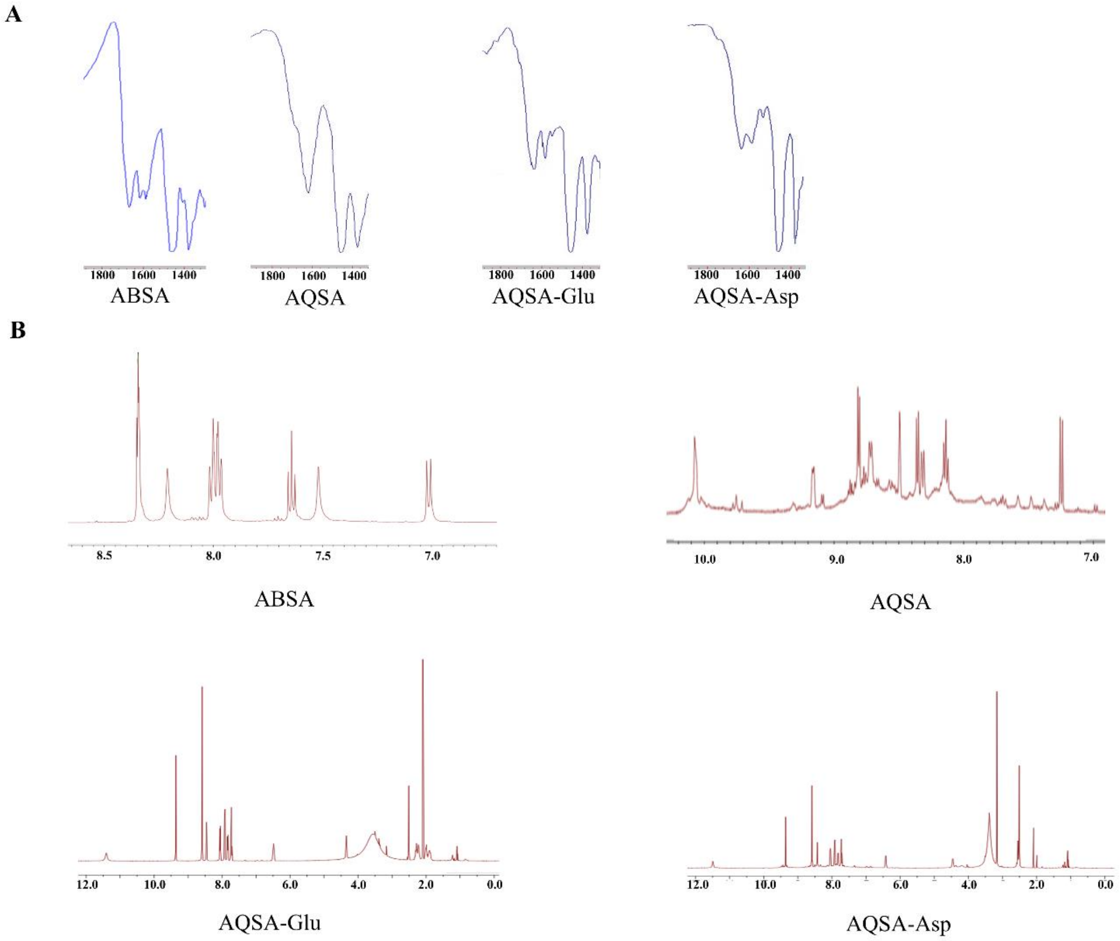

Supplement: Supplementary file 1 [file pharmaceutics-16-01546-s001.zip › pharmaceutics-3303784-supplementary.pdf]
